# Aberrant Expression of Functional BAFF-System Receptors by Malignant B-Cell Precursors Impacts Leukemia Cell Survival

**DOI:** 10.1371/journal.pone.0020787

**Published:** 2011-06-08

**Authors:** Sara Maia, Marc Pelletier, Jixin Ding, Yen-Ming Hsu, Stephen E. Sallan, Sambasiva P. Rao, Lee M. Nadler, Angelo A. Cardoso

**Affiliations:** 1 Department of Medical Oncology, Dana-Farber Cancer Institute, Harvard Medical School, Boston, Massachusetts, United States of America; 2 Department of Pediatric Oncology, Dana-Farber Cancer Institute, Harvard Medical School, Boston, Massachusetts, United States of America; 3 Division of Hematology/Oncology, Indiana University School of Medicine, Indianapolis, Indiana, United States of America; 4 Department of Protein Engineering, Biogen Idec, Cambridge, Massachusetts, United States of America; 5 Department of Immunobiology/Autoimmunity, Biogen Idec, Cambridge, Massachusetts, United States of America; 6 Institute of Molecular Medicine, Lisbon, Portugal; Vanderbilt University Medical Center, United States of America

## Abstract

Despite exhibiting oncogenic events, patient's leukemia cells are responsive and dependent on signals from their malignant bone marrow (BM) microenvironment, which modulate their survival, cell cycle progression, trafficking and resistance to chemotherapy. Identification of the signaling pathways mediating this leukemia/microenvironment interplay is critical for the development of novel molecular targeted therapies.

We observed that primary leukemia B-cell precursors aberrantly express receptors of the BAFF-system, BAFF-R, BCMA, and TACI. These receptors are functional as their ligation triggers activation of NF-κB, MAPK/JNK, and Akt signaling. Leukemia cells express surface BAFF and APRIL ligands, and soluble BAFF is significantly higher in leukemia patients in comparison to age-matched controls. Interestingly, leukemia cells also express surface APRIL, which seems to be encoded by APRIL-δ, a novel isoform that lacks the furin convertase domain. Importantly, we observed BM microenvironmental cells express the ligands BAFF and APRIL, including surface and secreted BAFF by BM endothelial cells. Functional studies showed that signals through BAFF-system receptors impact the survival and basal proliferation of leukemia B-cell precursors, and support the involvement of both homotypic and heterotypic mechanisms.

This study shows an unforeseen role for the BAFF-system in the biology of precursor B-cell leukemia, and suggests that the target disruption of BAFF signals may constitute a valid strategy for the treatment of this cancer.

## Introduction

Increasing evidence indicates that microenvironmental cues play critical roles in cancer biology and that malignant cells are responsive to multiple extrinsic factors from their microenvironment. These stimuli involve both soluble factors and receptor/ligand interactions, which mediate or influence processes as tumor development, maintenance, drug-resistance and immune evasion.[1] Studies indicate that the ‘leukemia microenvironment’ supports acute lymphoblastic leukemia (ALL) cells developing in the bone marrow (BM), namely by providing survival/proliferation signals and by functioning as potential niches for chemotherapy-resistant tumor cells.[2], [3], [4], [5], [6]

Ligands and receptors of the tumor-necrosis factor (TNF) superfamily play significant roles in B-cell development and homeostasis. B-cell-activating factor (BAFF; BLyS)[7] is a TNF-superfamily member expressed by various cell types [reviewed [8]], and has been shown to prolong B-cell survival.[9], [10] BAFF transgenic mice exhibit increased number of B-cells, expressing elevated levels of anti-apoptotic molecules.[11], [12] In addition to serving as a potent B-cell survival factor,[8] BAFF also functions as a costimulator of B-cell proliferation.[7], [13] BAFF shares significant homology with APRIL (a proliferation-inducing ligand).[8], [14]

Three known receptors for BAFF − BCMA (B-cell maturation antigen),[15], [16] TACI (transmembrane activator, calcium-modulator, cyclophilin ligand interactor)[16], [17] and BAFF-R (BAFF-receptor) have been identified, which are expressed by immature/mature B-lymphocytes.[18], [19], [20], [21] BCMA and TACI also bind APRIL, whereas BAFF-R exclusively interacts with BAFF. The role of BCMA in B-cell homeostasis remains undefined: whereas injection of BCMA-Ig, as decoy receptor, resulted in marked B-cell reduction in secondary lymphoid organs,[15] BCMA-deficient mice did not exhibit an obvious phenotype.[22], [23] TACI-null mice showed elevated B-cell numbers, suggesting a negative regulatory role for TACI on B-cell homeostasis;[24], [25] TACI-Ig administration also led to inhibition of T-cell-independent immune responses,[26] abolition of germinal center formation[27] and prolonged B-cell lifespan.[17] The phenotype of BAFF-R-deficient mice is similar to that of BAFF-deficient mice, with impaired B-cell maturation beyond the T1 stage, decreased Ig levels and decreased T-cell-dependent and T-cell-independent immune responses.[20], [21], [28] This suggests that the BAFF/BAFF-R axis is the main driver for B-cell survival and maturation.[20], [21], [28]

The mechanisms regulating BAFF-system molecule expression are poorly understood. Interleukin-10, Interferon-α (IFN-α), IFN-γ and CD154/CD40L can upregulate BAFF or APRIL expression in different cells, including macrophages/monocytes and dendritic cells.[29], [30] During malignant transformation, cells undergo genetic/epigenetic alterations that drive changes in their proteome, such as over-expression or aberrant expression of critical molecules. APRIL, which is expressed at low levels by normal cells,[29], [30] is upregulated on B-cell chronic lymphocytic leukemia (CLL), lymphoma and myeloma cells ([14], reviewed in [31]), i.e. malignancies involving late-stage B-cells. BAFF is expressed by malignant mature B-cells,[31] in contrast to their normal counterparts.[7] Both ligands have been implicated in proliferation and survival of malignant B-cells.[31]

The pattern of BAFF-system receptor expression during B-cell development[28] suggests that BAFF/APRIL lack functional roles in early stage B-cells. Fittingly, BAFF transgenic mice exhibited normal cell distributions and differentiation of precursor/progenitor B-lineage cells.[11], [12], [17] Here, we show that primary leukemia B-cell precursor ALL (B-ALL) express functional receptors of the BAFF-system, particularly BAFF-R, and that their stimulation by BAFF potentiates cell proliferation and results in the engagement of survival pathways. Moreover, we show that BAFF and APRIL are expressed by cells of the BM microenvironment known to support leukemia, as well as by the leukemia cells themselves. These studies indicate that BAFF-system ligands function through both homotypic and heterotypic mechanisms on leukemia B-cells, revealing a new role for the BAFF-system in B-ALL biology.

## Results

### Leukemia B-cell Precursors Express BAFF-R, BCMA, and TACI Receptors

**Figure 1:**
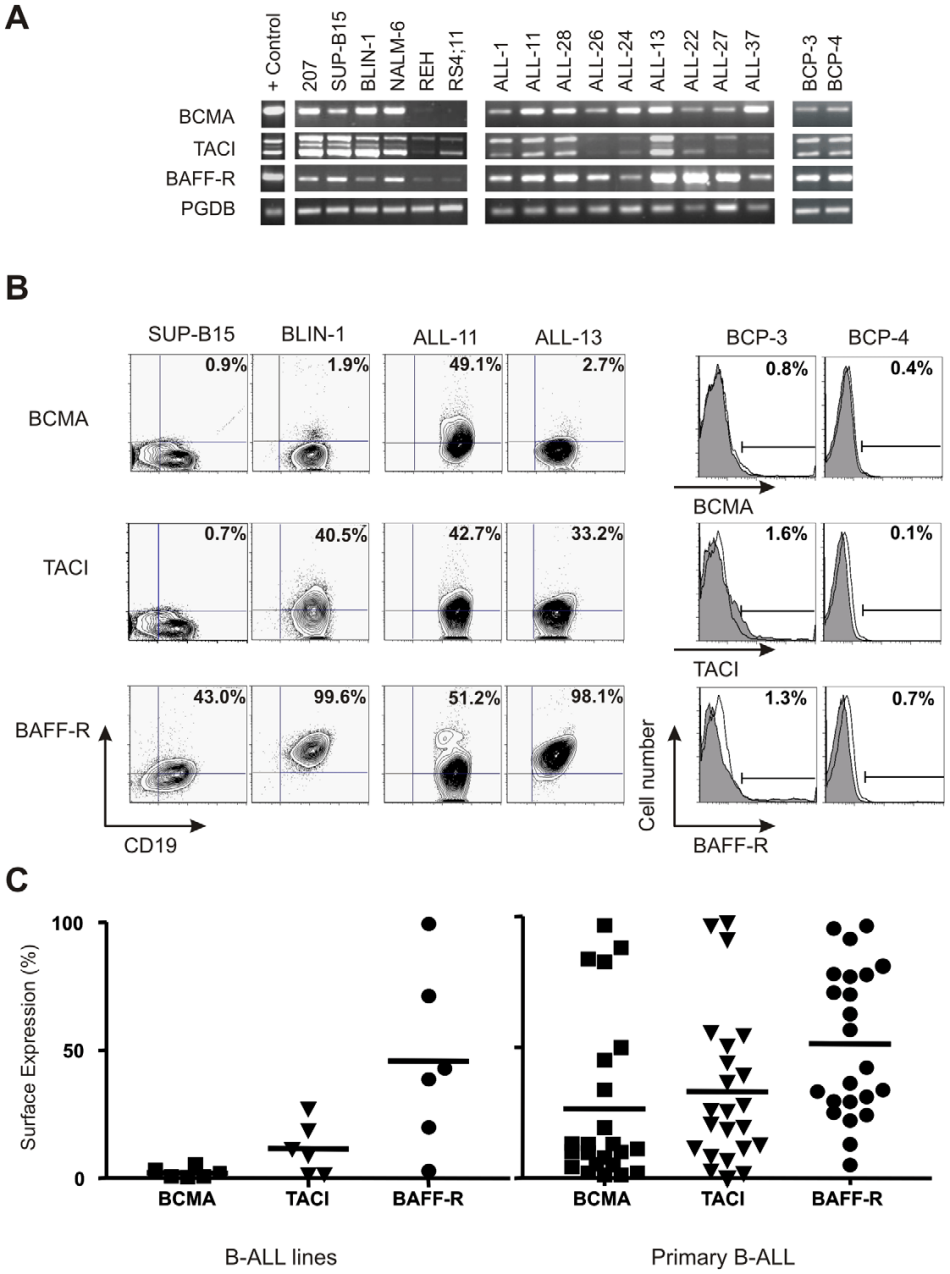
Primary B-ALL express BAFF-system receptors. (A) Electrophoretic analysis of RT-PCR products of *BCMA*, *TACI* and *BAFF-R* in B-ALL lines (left), primary B-ALL (center) and BCP from normal donors (right). Splenic mature B-cells as positive control (+); *PBGD* as control transcript. (B) Flow cytometry of surface BCMA, TACI and BAFF-R on representative B-ALL lines, B-ALL patients and normal BCP. (C) Summary of surface BCMA (▪), TACI (▾) and BAFF-R (•) expression (% of cells) in B-ALL patients (n = 23; BCMA+TACI+BAFF-R, 17/23; BCMA+BAFF-R 2/23; TACI+BAFF-R 3/23; only BAFF-R, 1/23; only TACI or BCMA, or BCMA+TACI, 0/23) and lines (n = 6) evaluated by flow cytometry; solid line indicates mean surface expression (% of cells). B-ALL lines: BCMA, mean 2.23%, range 0.6–5.54%; TACI, mean 11.67%, range 1.51–27.2%; BAFF-R, mean 45.96%, range 3.07–99.5%.

Little is known on the key microenvironmental factors involved in the biology of B-cell precursor ALL (hereafter referred as B-ALL; includes leukemias of pro-B, pre-B and immature B-cells). As part of a screening assessing the putative involvement of TNF-superfamily members on B-ALL, we assessed the expression of *BCMA*, *TACI* and *BAFF-R* in primary leukemia B-cell precursors (patients' characteristics in Table S1). PCR analyses of full-length mRNAs were performed in primary B-ALL cells (BM or PB; n = 36) and B-ALL lines (n = 6). *BAFF-R* and *TACI* transcripts were detected in all B-ALL lines, whereas *BCMA* was detected in 4 of them; their relative expression was stronger in pre-B-cell (207, SUP-B15, BLIN-1, NALM-6) than in pro-B-cell lines (REH, RS4;11; Figure 1A). Analysis of primary B-ALL specimens showed that *BCMA* and *BAFF-R* transcripts are present in all cases tested, with *TACI* mRNA detected in 35 patients (Figure 1A; data not shown). No differences were observed between BM and PB specimens from the same patient (n = 6; data not shown). Nucleotide sequencing confirmed that the transcripts amplified corresponded to the respective genes, and no mutations were detected (n = 12 patients; data not shown). *BCMA*, *TACI* and *BAFF-R* transcripts were observed also in normal B-cell precursor (BCP; containing pro-/pre-B and immature B-cells; n = 4) specimens from age-matched healthy donors (Figure 1A; data not shown).

Receptor expression was analyzed at a single cell level by flow cytometry in primary B-ALL (n = 23), B-ALL lines (n = 6) and normal BCP (n = 4) samples. Surface forms of BAFF receptors (Figure 1B, left and central panels for representative cases) were detected in B-ALL primary cells and cell lines. For the latter, BAFF-R was the receptor most commonly expressed whereas BCMA and TACI were generally low or absent (Figure 1C, left panel). As shown in Figures 1B and 1C (right panel), all B-ALL patients express BAFF-R (mean 51.46%, range 5.09–96.5%), TACI (mean 33.15%, range 0.11–97.7%), whereas BCMA was seen in some patients (mean 26.56%; range 1.28–96.7%). Individual analyses revealed that all B-ALL patients tested expressed at least one of these receptors (data not shown). Importantly, BAFF-system receptors are absent or weakly expressed on normal BCP (Figure 1B; BCMA, mean 1.15%, range 0.39–1.75%; TACI, mean 3.24%, range 0.05–7.9%; BAFF-R, mean 2.05%, range 0.07–4.9%). This is in accordance with reported studies showing that the expression of these receptors is restricted to more differentiated stages of B-cell lineage development.[18], [19], [20] The presence of mRNA transcripts (Figure 1A, right panel) but not surface receptors in normal BCP suggests their regulation via post-transcriptional mechanism(s) during normal B-cell development.

### B-ALL Cells Express BAFF and APRIL

**Figure 2:**
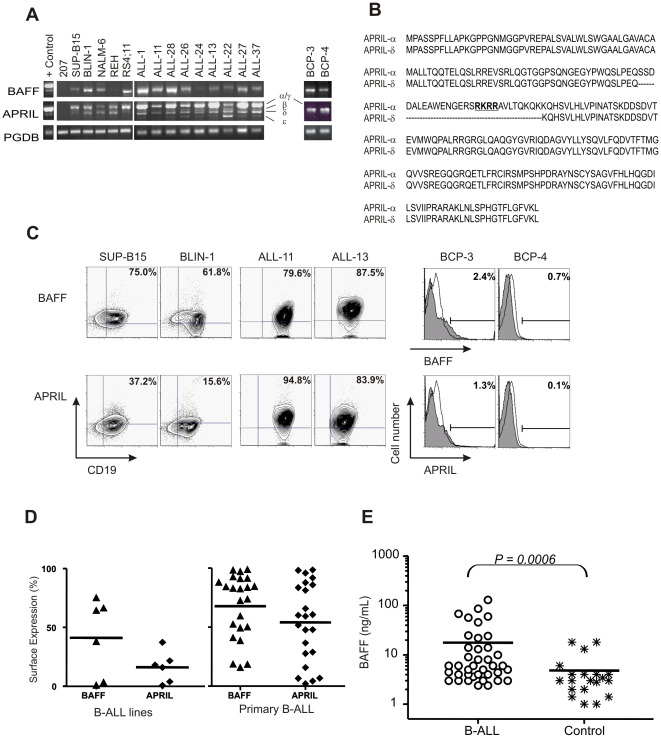
Primary B-ALL express BAFF and APRIL. (A) Electrophoretic analysis of RT-PCR products of *BAFF* and *APRIL* in B-ALL lines (left), primary B-ALL (center) and normal BCP (right). Monocytes as positive control (+); *PBGD* as control transcript. A 940bp band was identified as the new isoform APRIL-δ. (B) Comparison of aminoacid sequences of APRIL-α and the predicted for APRIL-δ, which results from alternative splicing with exon-1/exon-3 fusion (lacking residues as dashed lines); this results in a 104 predicted aminoacid protein lacking the motif for furin convertase (in bold). (C) Flow cytometry of BAFF, APRIL expression on representative cases of B-ALL patients, B-ALL lines and normal BCP. (D) Summary of BAFF (▴) and APRIL (♦) surface expression in B-ALL patients (n = 23; BAFF+APRIL, 22/23; only BAFF, 1/23; only APRIL, 0/23) or lines (n = 6) evaluated by flow cytometry; solid line indicates mean surface expression (% of cells). (E) ELISA for soluble BAFF in plasma from B-ALL patients (n = 39) or age-matched controls (n = 21); solid lines indicate mean levels of BAFF for each group. Statistical analysis performed using Mann-Whitney test.

We analyzed leukemia B-cells for expression of BAFF and APRIL. Most B-ALL lines (5 of 6) and primary B-ALL specimens (35 of 36) expressed *BAFF* transcripts, with variable intensity (Figure 2A; data not shown). No differences were observed between BM and PB specimens from the same patient (n = 6; data not shown). Nucleotide sequencing confirmed that the amplified product corresponded to *BAFF* (n = 3 patients; data not shown). *APRIL* mRNA was detected in all primary B-ALL and in most cell lines (5 of 6), with the presence of distinct transcripts, corresponding to isoforms *-α*, *-β* and *-γ* (Figure 2A), as confirmed by nucleotide sequencing. Interestingly, 35 of 36 primary B-ALL also exhibited an additional band, which nucleotide sequencing (n = 11) revealed as a novel *APRIL* isoform, *APRIL-δ* (reported to GenBank as accession number DQ149579) that results from an alternative-splicing event where exon 2 is missing, thus lacking the furin convertase motif RKRR [32] (Figure 2B; Figure S1). *BAFF* and *APRIL* transcripts were observed also in normal BCP (n = 4) specimens from age-matched healthy donors (Figure 2A; data not shown).

Analyses of surface expression of BAFF and APRIL on B-ALL primary cells (n = 23), cell lines (n = 6), and normal BCP (n = 4), showed that both ligands are expressed by malignant cells (Figure 2C, left and central panels for representative cases). All primary precursor B-ALL cases express BAFF, with a mean expression of 67.8% (Figure 2D; range 15.5–98.6%) and most express APRIL (Figure 2D; mean 54.03%, range 1.94–98.5%). Individual analyses revealed that cells from 22 out of 23 B-ALL patients expressed both BAFF and APRIL on their surface (data not shown). This finding was unexpected since membrane-bound APRIL is efficiently processed into its soluble form.[8], [32] Since the novel isoform APRIL-δ lacks the consensus motif required for furin convertase-mediated cleavage (Figure 2B) and contains a transmembrane-like domain, it is possible that the APRIL detected on the cell surface of B-ALL cells represents this isoform. As seen with BAFF-system receptors, the expression of the ligands was also almost undetectable on normal BCP (Figure 2C right panel for representative cases; BAFF, mean 1.42%, range 0.03–2.94%; APRIL, mean 2.59%, range 0.07–5.33%).

Finally, the presence of soluble BAFF in the leukemia milieu was assessed by ELISA in the plasma of B-ALL patients (n = 39) and compared to the plasma of age-matched controls (n = 21). Mean levels of soluble BAFF are significantly higher in B-ALL patients in comparison to control specimens (Figure 2E; p = 0.0006; B-ALL, mean 17.68 ng/mL, range 2.4–130.0 ng/mL; controls, mean 4.81 ng/mL, range 1.0–18.0 ng/mL).

### BM Microenvironment Cells Express BAFF and APRIL

**Figure 3:**
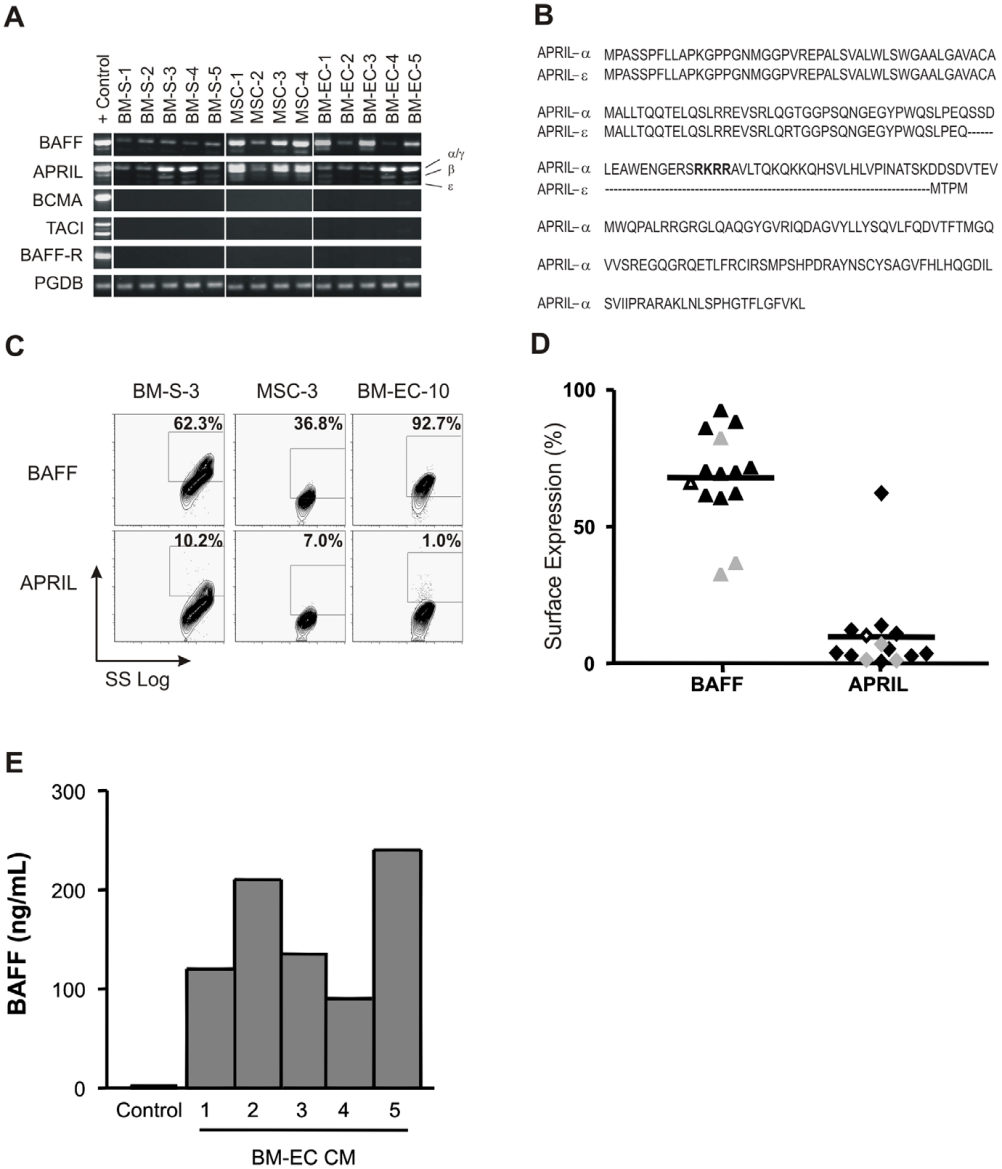
BAFF and APRIL expression in BM microenvironmental cells. (A) Electrophoretic analysis of RT-PCR products of *BAFF* and *APRIL* in BM-S, BM-EC and MSC. Monocytes and splenic mature B-cells used as positive controls (+) for BAFF-system ligands and receptors, respectively; *PBGD* as control transcript. (B) Comparison of aminoacid sequence of *APRIL*-α and *APRIL*-ε, lacking residues as dashed lines), which results from alternative splicing with exon-1/exon-4 fusion; predicted 90 aminoacid protein lacking the furin convertase motif (in bold) and with early stop codon. (C) Flow cytometry of BAFF, APRIL expression in representative BM-S, MSC and BM-EC cases. (D) Summary of BAFF (Δ) and APRIL (◊) surface expression (%) in BM microenvironmental cells (n = 14; symbols: BM-S, open; MSC, gray; BM-EC, black). Solid line indicates mean surface expression (% of cells), as assessed by flow cytometry. (E) ELISA for soluble BAFF in CM from BM-EC cultures (n = 5 biological replicates).

BM-stromal (BM-S) myofibroblasts support leukemia cell functions[2], [3] and tumor-stimulated BM endothelium (BM-EC) promotes the survival of primary leukemia cells**,** [6] with BM endothelial microdomains functioning as niches for leukemia cell maintenance.[33] Since BAFF and APRIL can promote malignant cell survival and proliferation in mature B-cell tumors developing in the BM,[31] we investigated the expression of BAFF-system transcripts in BM-EC (n = 5), mesenchymal stem cells (MSC; n = 4) and BM-S (n = 5). Both *BAFF* and *APRIL* mRNA were detected in all BM microenvironment cell types tested, but not transcripts for the BAFF-system receptors (Figure 3A). In some specimens, a smaller *APRIL* transcript was observed (Figure 3A, lower band); sequencing analysis showed that it represents a novel alternative-spliced isoform – *APRIL*-ε, missing exons 2 and 3, and containing an early stop codon (Figure 3B; deposited as GenBank EF211088).

Expression of surface BAFF and APRIL on these cell populations was assessed by flow cytometry; illustrative cases are shown in Figure 3C and the overall data summarized in Figure 3D. BAFF is expressed on most of these cells, particularly on BM-EC (mean expression 68.01%; range 32.7–92.7%). In contrast, with exception of 1 donor, expression of surface APRIL was low to undetectable (mean expression 9.8%; range 0.6–62.2%), which may be due to the lack of *APRIL-δ* transcripts. Finally, ELISA analyses showed the presence of soluble BAFF in conditioned medium (CM) from BM-EC cultures (Figure 3E; range: 90–240ng/mL). Taken together, the expression pattern of BAFF-system ligands and receptors on leukemia B-cells and BM microenvironment cells raises the hypothesis that signals involving this molecular axis may occur through both homotypic and heterotypic mechanisms.

### BAFF and APRIL Bind to Leukemia B-Cells Expressing BAFF-System Receptors

**Figure 4:**
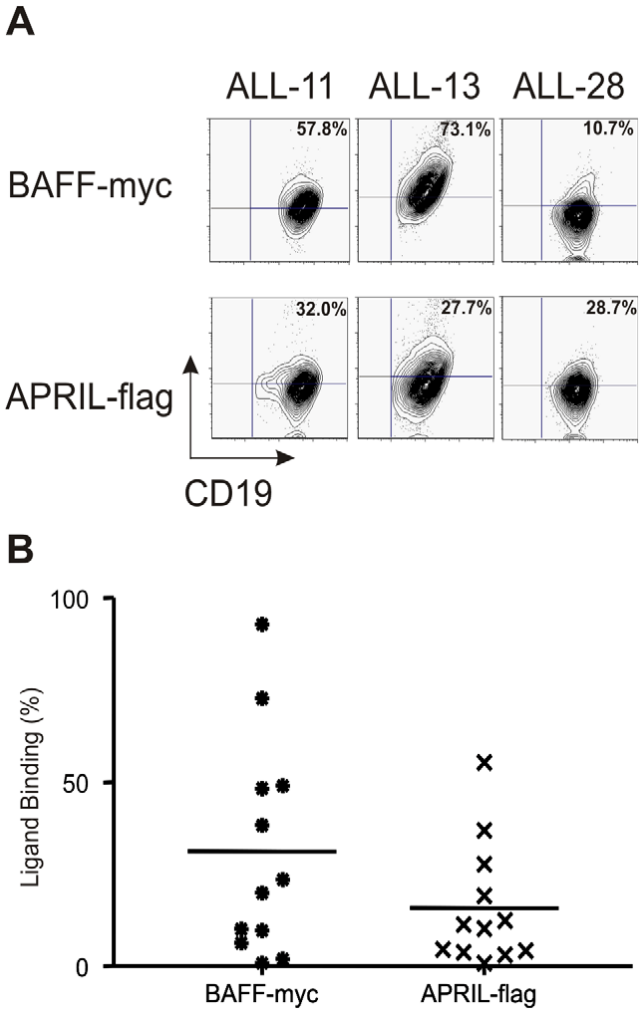
BAFF and APRIL bind to primary B-ALL cells. (A) Flow cytometry analyses of BAFF and APRIL binding to primary B-ALL cells, on three representative patients. (B) Summary of BAFF (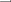) and APRIL (x) binding to primary B-ALL cells (n = 12); solid line represents the mean binding of the respective ligands (% of cells), as assessed by flow cytometry.

To investigate whether the BAFF-system receptors expressed on B-ALL cells are functional, we first performed binding assays on primary B-ALL cells (n = 12) using human BAFF-myc or APRIL-flag fusion proteins. It was previously demonstrated that these fusion proteins specifically bind to BCMA, TACI and/or BAFF-R.[7], [34], [35] Figure 4A depicts three representative cases, showing variable levels of BAFF-myc or APRIL-flag binding to primary leukemia cells. In most patients studied, BAFF-myc binds to at least 25% of the leukemia cells (Figure 4B; mean 31.1%, range 0.8–92.9%), whereas binding of recombinant APRIL seems to be less pronounced (Figure 4B; mean 15.76%, range 0.8–55.3%).

### BAFF Ligation to B-ALL Engages NF-κB, MAPK, and PI3K/AKT Signaling Pathways

**Figure 5:**
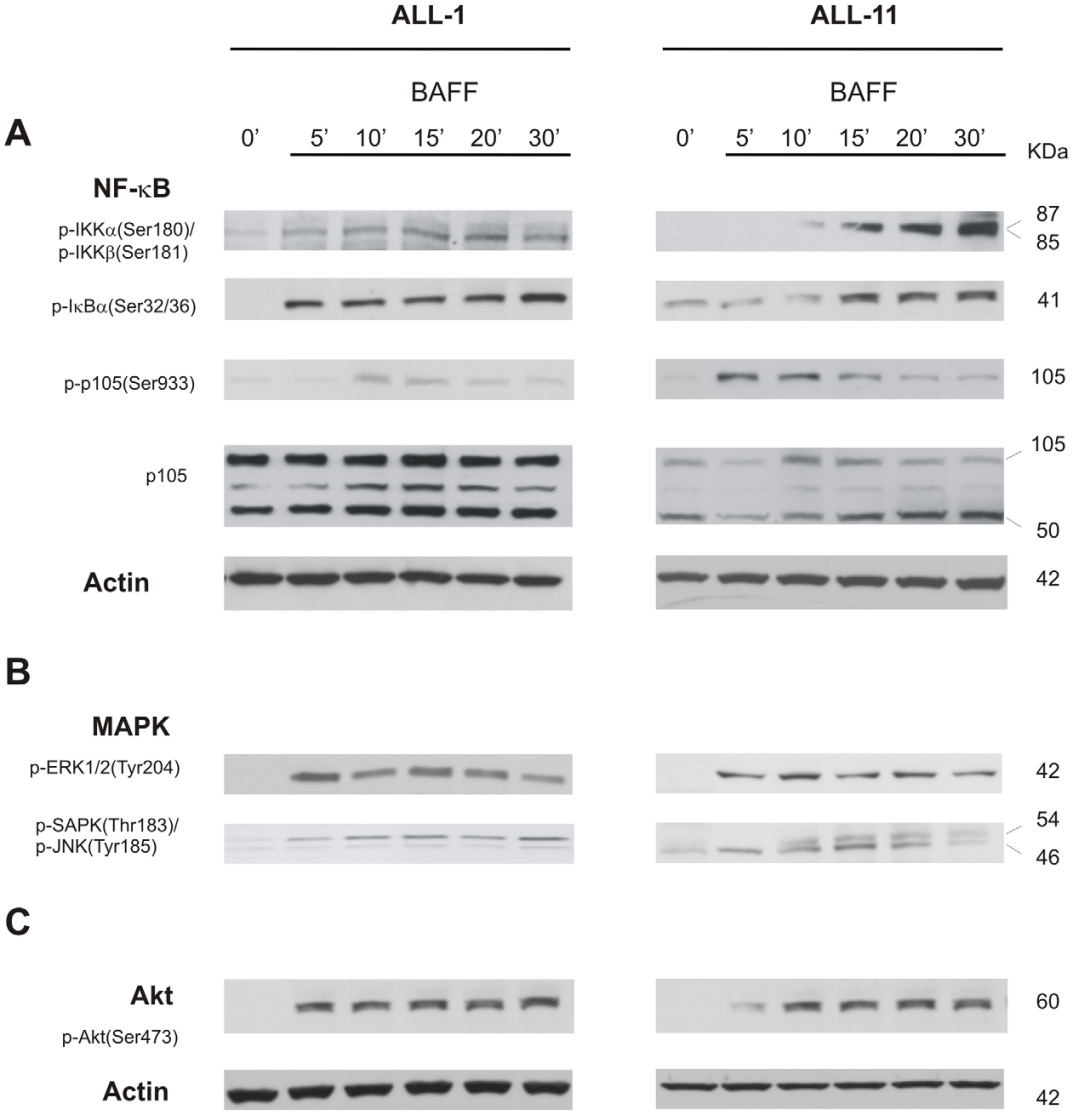
BAFF activates NF-κB, MAPK, and Akt signaling in primary B-ALL. (A) BAFF-triggered NF-κB activation was assessed by immunoblotting using antibodies for p-IKKα/p-IKKβ, p-IκBα, p-p105, the precursor form of p105 and its activated form p50. (B) MAPK activation by assessing the phosphorylation status of ERK1/2 and SAPK/JNK. (**C**) PI3K/Akt activation assessed using antibody for p-Akt.

To demonstrate the functional integrity of BAFF-system receptors in B-ALL cells, we evaluated whether their ligation triggered the activation of NF-κB, MAPK, and PI3K/Akt signaling, which are critical events on survival and proliferation of malignant B-cells.[36], [37], [38] Since BAFF-R was the most frequently expressed receptor (Figure 1), the ensuing studies were performed using BAFF. Primary B-ALL cells (n = 3) were stimulated with BAFF-myc for different periods (5–30 minutes), and were analyzed by immunoblotting for activation status of IKKα/β, IKBα, p105, ERK1/2, JNK/SAPK and Akt. BAFF ligation triggered NF-κB signaling in leukemia B-cells, as shown by the phosphorylation of the catalytic subunits IKKα/IKKβ, the inhibitory protein IKBα, and the transcription factor p105/NF-κB1 (Figure 5A), and subsequent augmentation of p50. BAFF ligation to B-ALL cells also triggered the activation of MAPK signaling, with the phosphorylation of ERK1/2 and JNK/SAPK kinases (Figure 5B), as well as PI3K/Akt signaling, with phosphorylation of Akt (Figure 5C). These observations demonstrate that primary B-ALL cells express functional BAFF-system receptors, whose ligation effectively triggers the activation of critical signaling transduction cascades.

### BAFF Potentiates Mitogenic Stimuli to B-Cell ALL

**Figure 6:**
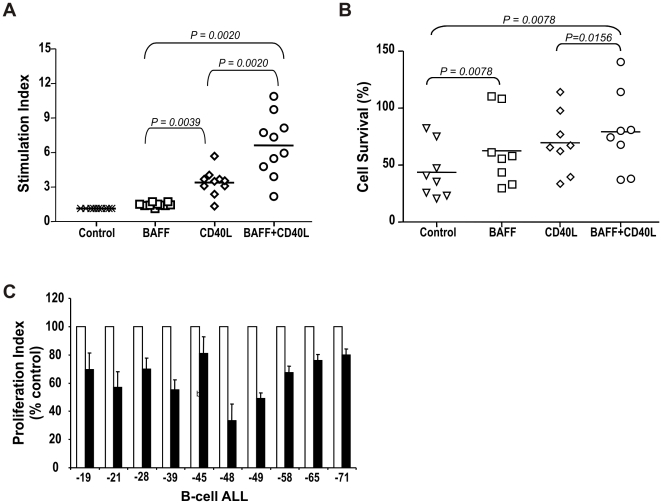
BAFF potentiates proliferation and mediates survival of primary B-ALL cells. (A) Proliferation of primary B-ALL cells (n = 10) cultured in medium alone (Control, x), BAFF-myc (□), CD40L (◊) or BAFF-myc+CD40L (○), as measured by thymidine incorporation. Each point corresponds to mean stimulation index (i.e, proliferation of test condition divided by proliferation in control medium) for each patient tested, per condition; solid line indicates the mean stimulation index per experimental condition. Statistical analysis performed using the Wilcoxon test. (B) Primary B-ALL (n = 8) cultured in control medium (Δ) or with BAFF-myc (□), CD40L (◊) or BAFF-myc+CD40L (○), and evaluated for cellular viability. Each point corresponds to mean survival percentage for each patient tested, per condition; solid lines indicate mean survival percentage per experimental condition. Statistical analysis performed using the Wilcoxon test. (C) Primary B-ALL cells (n = 10) cultured with BCMA-Fc (black bars) or control fusion protein (white bars). Experiments performed in triplicate, and results expressed as mean proliferation index (i.e, proliferation in BCMA-Fc divided by proliferation in control-Fc) for BCMA-Fc vs. control fusion protein.

To evaluate the impact of BAFF stimulation on B-ALL functions, primary B-ALL (n = 10) were cultured for 72 h in control medium or in the presence of BAFF-myc and/or CD40L, as we and others have reported that CD40 ligation is mitogenic for B-ALL cells.[39], [40] BAFF alone did not promote substantial cell proliferation (Figure 6A; mean 1.34, range 1.0–1.59-fold increase) whereas, as expected, CD40L was mitogenic for B-ALL cells in most cases (Figure 6A; mean 3.26, range 1.19–5.54-fold increase). Importantly, BAFF significantly potentiated the mitogenic effect of CD40L, with 4.84-fold increase in cell proliferation in comparison to BAFF alone (Figure 6A; mean 6.48, range 2.06–10.76; p = 0.002 vs. BAFF alone; p = 0.002 vs. CD40L alone). These results indicate that B-ALL cells are functionally responsive to signals through BAFF-system receptors, and that BAFF potentiates the proliferative response of leukemia B-cells to mitogenic stimuli, such as CD40 crosslinking.

### BAFF Potentiates B-ALL Cell Survival

To assess BAFF role on B-ALL cell survival, primary B-ALL samples (n = 8) were cultured for 24h in control medium or in the presence of BAFF-myc and/or CD40L. BAFF alone improved ALL cell survival (Figure 6B, mean 62.51%, range 29.67–110.3%; p = 0.0078 vs. control); as expected, CD40L stimulation increased leukemia cell viability in comparison to control conditions (Figure 6B, mean 69.57%, range 33.6–114.0%; p = 0.0078 vs. control), without significant differences between these two conditions (Figure 6B, p = 0.1484). Interestingly, BAFF seems to potentiate the survival effect of CD40L in comparison with CD40L alone (Figure 6B, mean 79.22%, range 37.21–140.6%, p = 0.0156 vs. CD40L alone). Using a soluble BCMA-Fc fusion protein with high-affinity for BAFF and APRIL, preventing their ligation to their cognate receptors on target cells,[41] we observed that BCMA-Fc inhibits or abrogates the survival effects of BAFF in leukemia B-ALL cells (Figure S2).

### Basal B-cell ALL Proliferation Involves Homotypic BAFF-System Signals

Since leukemia cells also express BAFF and to a lesser extent APRIL, we evaluated whether homotypic or autocrine mechanism(s) involving these molecules may operate in B-cell ALL. Functional studies using the soluble BCMA-Fc fusion protein,[41] showed marked inhibition of the ‘basal’ proliferation of primary B-ALL cells (n = 10), in all cases tested (Figure 6C; mean 62.03%, range 33.73–78.58%; p<0.0001 BCMA-Fc vs. control Fc). This study suggests the involvement of BAFF-system ligands expressed or secreted by leukemia B-cells in homotypic interactions and/or autocrine loops mediating B-ALL cell functions.

## Discussion

There is increasing interest in dissecting the microenvironmental cues that critically impact leukemia cell functions in the malignant BM. We observed that leukemia precursor B-cells aberrantly express BAFF-system receptors, and that their cognate ligands BAFF and APRIL are expressed in the BM microenvironment, as well as by leukemia cells. A recent study reported the expression of BAFF-R in B-ALL cells and showed that BAFF stimulation supported the survival of leukemia cells, attenuating the cytotoxic effects of the kinase inhibitor nilotinib in Ph-positive leukemias.[42] Our work on a larger dataset of patients shows that in addition to functional BAFF-R, B-ALL cells also express TACI and BCMA, and that virtually all patients express at least one of the BAFF-system receptors. More importantly, we show increased BAFF levels in the leukemic BM, and provide evidence supporting the involvement of both homotypic and heterotypic signals through BAFF-system receptors in mediating B-ALL survival. Finally, we demonstrate that blockade of these signals using a BCMA-Fc decoy markedly inhibit or abrogate the effects of BAFF signals in B-ALL cell survival.

The expression of functional BAFF-system receptors by B-ALL was unexpected since their physiological expression seemed restricted to later B-cell lineage developmental stages.[28] We confirmed the lack of BCMA, TACI and BAFF-R proteins in normal BCP despite detection of their respective transcripts, suggesting post-transcriptional regulation of receptor expression in early B-cell development. BCMA protein is seen mainly in mature B-cells, whereas TACI and BAFF-R are first detected on immature B-cells.[28] In humans, BAFF-R is expressed first in immature B-cells, and BCMA and TACI in germinal center B-cells; other study reported that, in the BM, plasma cells express BCMA and TACI, but not BAFF-R.[43] Studies in mice null for individual BAFF-system receptors suggest that they lack significant physiological roles in early B-cell development.[8] The administration of BCMA-Ig (as decoy receptor) resulted in marked reduction of B-cells in all secondary lymphoid organs,[15] suggesting that while BCMA is dispensable, its ligands are critical for B-cell survival and maintenance. TACI-deficient mice displayed elevated B-cell numbers, suggesting a role as negative regulator of B-cell homeostasis.[24], [25] The phenotype of BAFF-R-deficient mice was similar to that of BAFF-null mice, suggesting that the BAFF-R-BAFF axis is the main driver for B-cell survival and maturation.[20], [21], [28]

Our data suggests that the malignant transformation of BCP results in the deregulation of mechanism(s) mediating the post-transcriptional control of BAFF-system receptor expression; it is unknown whether this deregulation is driven by genetic or epigenetic factors associated with BCP transformation or is a response to microenvironment cues in the leukemic BM. BAFF-R can be positively regulated by B-cell receptor stimulation and Toll-like receptor (TLR)-associated signaling, and negatively regulated by TNFR-associated factor-3; TLR signals also upregulate TACI.[44], [45], [46] Although TLR mRNAs (*TLR1* to *-7*) were detected in leukemia lines and TLR9 protein in primary B-ALL, and B-ALL are responsive to TLR stimulation by CpG oligodeoxynucleotides,[47], [48] there is no evidence supporting a role for TLR signals within the leukemic BM, or their effects in B-ALL biology.

Previous studies identified the malignant microenvironment as the main source of BAFF-system ligands in BM cancers. In myeloma, it has been shown BAFF/APRIL secretion by monocytes, neutrophils, and osteoclasts (only APRIL), but not by stromal cells;[49] other study showed BAFF surface expression and increased levels of soluble BAFF and APRIL in supernatants of patient-derived stroma in comparison to stromal cells from normal donors.[50] In CLL, leukemia-supporting nurse-like cells express high BAFF and APRIL levels, which seem to mediate leukemia cell survival.[38] Our observation that BM-EC express and secrete BAFF is interesting, as studies suggest an important role for BM endothelial niches on leukemia cell survival[6], [33], and for the regulation of normal, and possibly, leukemia stem cells. It would be interesting to investigate whether BAFF-/APRIL-rich areas in the BM (as seen for plasma B-cells),[51] are involved in regulating B-ALL cells with leukemia-initiating properties. The expression of BAFF/APRIL by leukemia BCP suggests the involvement of BAFF-system signaling, via cell-cell contact and/or through autocrine mechanisms. BAFF and APRIL expression was reported in other B-cell malignancies, namely non-Hodgkin's lymphoma, plasma-cell leukemia and Waldenstrom's macroglobulinemia;[8], [31], [51] APRIL as a soluble factor, whereas BAFF was detected both as soluble and membrane form.[8] Here, we identified a new *APRIL* isoform, *APRIL-δ*, lacking the consensus motif for furin convertase-mediated cleavage,[32] which may explain the surface APRIL seen in B-ALL cells. Analyses of genomic sequences (introns 1–2) showed canonical splicing donor and acceptor sites in the human gene and in other species (Figure S3). In addition to soluble BAFF, which is elevated in patients' plasma, leukemia B-cells express membrane BAFF and blockade with BCMA-Fc markedly inhibited basal leukemia cell proliferation, further supporting the involvement of homotypic interactions on the functional role of the BAFF-system in B-ALL.

The B-ALL-expressed BAFF-system receptors are functional as they bind BAFF and/or APRIL and their ligation triggers NF-κB, MAPK, and Akt signaling, mediating leukemia cell survival and potentiating their response to CD40L mitogenic signals. NF-κB and MAPK activation was expected, and sheds light on molecular mechanisms by which BM microenvironmental cues, or at least extrinsic signals, may impact on leukemia BCP. Studies in other B-cell malignancies (lymphoma, myeloma and CLL) showed the engagement of NF-κB, MAPK, and Akt by BAFF or APRIL stimulation.[36], [37], [38] Our study unveils the involvement of new molecular axis in the biology of malignant BCP, particularly in the crosstalk between leukemia cells and their supportive BM microenvironment.

We observed BAFF-system spliced isoforms in B-ALL cells, including a *TACI* isoform (GenBank AY302137), *APRIL-β* (NM_172087), *APRIL-γ* (NM_172088), and the newly identified *APRIL-δ* and *APRIL-ε* (GenBank DQ149579 and EF211088, respectively). *APRIL-ε* was also seen in microenvironmental cells. It is unknown if these isoforms are translated into functional proteins, and whether they may trigger signals on leukemia B-cells or alter their responsiveness to BAFF/APRIL signals (such as decoy receptors). We observed that a novel *BCMA* isoform is secreted as a soluble receptor that effectively inhibits BAFF signals in mature B-cells (Maia *et al*., manuscript submitted), but have no evidence that it may affect the effects of BAFF/APRIL on leukemia cells within the BM microenvironment.

The involvement of BAFF and APRIL in B-ALL biology offers new and unanticipated molecular target(s) for this cancer. Several therapeutic agents (as the anti-BAFF antibody Lymphostat-B, the BAFF-antagonist AMG-623, and decoy fusion proteins TACI-Fc/Atacicept, BCMA-Fc, BR3-Fc) are being assessed for the targeted disruption of the BAFF-system in B-cell disorders and hematological malignancies.[8], [31] Studies are necessary to define the critical role of BAFF-system-triggered signals in B-ALL development, and to validate BAFF-system targeting as a valid strategy for the treatment of B-ALL patients.

## Materials and Methods

### Ethics Statement

Appropriate written informed consent was obtained and studies performed in accordance with the Declaration of Helsinki, and under a protocol approved by the Dana-Farber Cancer Institute's Institutional Review Board.

### Primary Cells and Cell Lines

Leukemia or normal specimens were collected according to institutional guidelines. B-ALL cells (diagnostic; >90% blasts) were collected from patients' BM or peripheral blood (PB) and included 21 BM, 22 PB and 6 BM+PB samples; mononuclear cells (MNC) were isolated by density centrifugation, and used for phenotypic, signaling and functional studies. Normal B-cell precursors (BCP; CD19^+^Igκ^-^/Igλ^-^) were selected from BM of 4 age-matched healthy donors using fluorescence-activated-cell-sorting (Coulter EpicsALTRA). BM microenvironmental cells (BM-EC; MSC; BM-S) were isolated from healthy donor's BM aspirates: BM-EC were purified using CD105 microbeads (Miltenyi-Biotech, Auburn, CA), and selected by culturing in EGM-2 (containing bFGF, VEGF, EGF and IGF-1; Cambrex, Walkersville, MD) [6], [52]; MSC isolated from the CD105^+^ fraction and selected by culture in MSCGM (Cambrex); BM-S cells prepared from the adherent fraction of MNC, through culture in MyeloCult™ media (Stem Cell Technologies, Vancouver, Canada), and were mainly composed of myofibroblasts. B-ALL cell lines 207, REH, SUP-B15, NALM-6 and RS4;11 were obtained from ATCC (Manassas, VA) or DSZM (Braunschweig, Germany), and BLIN-1 from Dr. Tucker LeBien (Univ. Minnesota); cells were tested prior use by flow cytometry to confirm their phenotype and developmental stage. Leukemia cells (lines, patients) were cultured in RPMI supplemented with 10% FBS (hereafter referred as RPMI-FBS).

### RT-PCR and Sequencing

Total RNA was purified using TRIZOL (Invitrogen, Carlsbad, CA), and cDNA prepared by reverse transcription at 42°C in a mix containing Improm II (Promega, Madison, WI) and pd(N) hexamers (Amersham). PCR amplifications of *BCMA*, *TACI*, *BAFF-R*, *APRIL* and *BAFF* were performed as described in Table S2. Amplification of the housekeeping gene *PBGD* was used as control. Specific bands were extracted, purified (QIAquick kit, Qiagen, Valencia, CA), and analyzed in an automatic sequencer (Applied Biosystems, Foster City, CA).

### Flow Cytometry Analyses

After Fc receptor blockade, cells were incubated with antibodies for: BCMA (R&D, Minneapolis, MN), BAFF (eBioscience, SanDiego, CA), TACI, BAFF-R or APRIL (Biogen Idec, Cambridge, MA), followed by fluorochrome-labeled secondary antibodies (Southern Biotechnology, Birmingham, AL). In B-ALL cells, CD19 was used as a B-cell marker (Beckman-Coulter, Miami, FL). As control, appropriate irrelevant isotype-matched antibodies were used. Cells were analyzed using a Cytomics™ FC500 cytometer (Beckman-Coulter) (at least 20,000-gated events acquired); data was analyzed using FlowJo (Tree Star, Ashland, OR).

### BAFF and APRIL Binding Assay

B-ALL cells were incubated with BAFF-myc or APRIL-flag fusion proteins (Biogen), washed, incubated with anti-myc (Santa Cruz Biotechnology, Santa Cruz, CA) or anti-flag M2 (Sigma, St. Louis, MO) antibodies, respectively, followed by goat-anti-mouse Ig-RPE antibodies. An irrelevant human Fc fusion protein was used as control. Samples were acquired using Cytomics™ FC500 and analyzed using FlowJo.

### Proliferation and Viability Assays

Viable primary B-ALL cells were cultured in RPMI-FBS (2×10^6^/mL) in 96-well plates for 72 h, in the presence of sCD40L (500 ng/mL; PeproTech, NJ), BAFF-myc (100 ng/mL; Biogen), BCMA-Fc (10 µg/mL; R&D), or control irrelevant fusion proteins, in the conditions indicated. Tritiated-thymidine (1 µCi/well) was added for 18 h prior to cell harvest, and thymidine incorporation determined using a Wallac Microbeta counter (Wallac-Oy, Turku, Finland). All conditions were tested in triplicate.

For viability assay, primary B-ALL cells were cultured in RPMI-FBS (2−3×10^5^ cells/well) in 96-well plates for 24 h, in the presence of sCD40L (500 ng/mL; PeproTech), BAFF-myc (100 ng/mL; Biogen), BCMA-Fc (10 µg/mL; R&D), or control irrelevant fusion proteins, in the conditions indicated. CellTiter-Glo luminescent cell viability assay (Promega, Madison, WI) was used. All conditions were tested in triplicate.

### Immunoblotting

Primary B-ALL cells were stimulated with BAFF-flag (100 ng/mL; Biogen) for the periods indicated, and lysates prepared in RIPA buffer. Immunoblotting was performed using antibodies for: p-IKKα(Ser180)/IKKβ(Ser181), p-IκBα(Ser32), p-p105(Ser933), p105, p-AKT(Ser473), p-JNK/SAPK(Thr183/Tyr185) (Cell Signaling, Danvers, MA); p-ERK1/2(Tyr204) (Santa Cruz); Actin (Sigma); and HRP-conjugated secondary antibodies (Promega).

### BAFF Quantification

Plasma from B-ALL patients and age-matched healthy donors was collected at diagnosis. After specimen centrifugation were filtered and kept at –70°C. Plasmas were diluted (1∶10) in PBS-1%BSA and precleared using protein A–Sepharose beads (10%v/v; Amersham). For conditioned media (CM) preparation, BM-EC were cultured to sub-confluence in EGM2. After washing with PBS, cells were cultured in serum/cytokine-free EBM2 media with, 0.5% BSA. CM were collected after 72 h, filtered and used immediately or stored at −70°C.

BAFF quantification was performed by ELISA with anti-BAFF capture and detection antibodies. Briefly, ELISA plates were coated overnight with anti-BAFF Buffy-5 antibody (2 µg/mL; Biogen); after blocking, serial dilutions of precleared sera were added, followed by a biotin-conjugated mouse-anti-human BAFF (0.5 µg/mL; Biogen). Detection was performed using alkaline phosphatase-conjugated streptavidin (Jackson ImmunoResearch) and appropriate substrate (Sigma). Plates were read at 405 nm, with standard curves generated using known quantities of rhBAFF, diluted in human serum and treated as described above for patients' samples.

### Statistical Analysis

Statistical significance was determined using the two-tailed non-parametric Mann-Whitney and Wilcoxon tests, using the Graphpad Prism-4.0 software (GraphPad, San Diego, CA). Differences were considered statistically significant when p≤0.05.

## Supporting Information

Figure S1 **Sequence alignment of human APRIL-α and APRIL-δ proteins.** The accession numbers of the sequences on the NCBI database are shown; NP_003799.1 corresponds to APRIL-α protein, and the ABA39072.1 sequence corresponds to the predicted APRIL-δ protein encoded by the exon-2-lacking spliced isoform identified in our B-ALL samples (DQ149579.1). The sequence NP_001185551.1 corresponds to the APRIL variant delta recently deposited by the DREAM investigators (mRNA accession number NM_001198622.1). The yellow box indicates the furin convertase motif RKRR, involved in the proteolytic cleavage of APRIL. Sequence analyses and alignments were performed using the ClustalW2 algorithm, for multiple sequence alignment [Chenna R, Sugawara H, Koike T, Lopez R, Gibson TJ, et al. (2003) Multiple sequence alignment with the Clustal series of programs. Nucleic Acids Res 31: 3497–3500.]. Below the alignment, a consensus line indicates: identical residues in all sequences (*).
(DOC)

Figure S2 **BCMA-Fc inhibits or abrogates the survival effect of BAFF on primary B-ALL cells.** Leukemia cells (n = 3) were cultured in control medium (white bars), with BAFF-myc (100 ng/mL; gray bars) or with BAFF-myc (100 ng/mL) plus BCMA-Fc (10 µg/mL; black bars). ATP levels were quantified at 24 h and results expressed as mean Survival Index, compared to cell viability measured at day 0 (set as 100%).
(DOC)

Figure S3 **Genomic sequences of the** ***APRIL*** **gene, showing the exonic-intronic boundaries (exon 1 to exon 3).** Nucleotides of exons are show in red, whereas intronic sequences are shown in black. The splicing sites donor GT and acceptor AG are indicated by the yellow or green boxes, respectively. Species abbreviations: Hs, *Homo sapiens*; Mm, *Mus musculus.* Rn, *Rattus norvegicus*; Bt, *Bos taurus*. Sequences were collected from the NCBI database, and the respective accession numbers are indicated.
(DOC)

Table S1 **Clinical characteristics of B-cell ALL patients (n = 72) used in this study.** Abbreviations: #, number; WBC, white blood cell count.
(DOC)

Table S2 **Oligonucleotides and PCR conditions used for the amplification of *BCMA*, *TACI*, *BAFF-R*, *APRIL*, *BAFF* and *PBGD*.**
(DOC)
