# Supplementary material for: Aberrant Expression of Functional BAFF-System Receptors by Malignant B-Cell Precursors Impacts Leukemia Cell Survival
Source: PLoS One. 2011 Jun 8;6(6):e20787. doi: 10.1371/journal.pone.0020787 (PMC3110793; doi:10.1371/journal.pone.0020787)
Supplement: Figure S1 — Sequence alignment of human APRIL-α and APRIL-δ proteins. The accession numbers of the sequences on the NCBI database are shown; NP_003799.1 corresponds to APRIL-α protein, and the ABA39072.1 sequence corresponds to the predicted APRIL-δ protein encoded by the exon-2-lacking spliced isoform identified in our B-ALL samples (DQ149579.1). The sequence NP_001185551.1 corresponds to the APRIL variant delta recently deposited by the DREAM investigators (mRNA accession number NM_001198622.1). The yellow box indicates the furin convertase motif RKRR, involved in the proteolytic cleavage of APRIL. Sequence analyses and alignments were performed using the ClustalW2 algorithm, for multiple sequence alignment [Chenna R, Sugawara H, Koike T, Lopez R, Gibson TJ, et al. (2003) Multiple sequence alignment with the Clustal series of programs. Nucleic Acids Res 31: 3497–3500.]. Below the alignment, a consensus line indicates: identical residues in all sequences (*). (DOC) [file pone.0020787.s001.doc]

**Figure S1**

Hs| NP_003799.1 MPASSPFLLAPKGPPGNMGGPVREPALSVALWLSWGAALGAVACAMALLT 50

Hs| NP_001185551.1 MPASSPFLLAPKGPPGNMGGPVREPALSVALWLSWGAALGAVACAMALLT 50

Hs| **ABA39072.1** MPASSPFLLAPKGPPGNMGGPVREPALSVALWLSWGAALGAVACAMALLT 50

**************************************************

Hs| NP_003799.1 QQTELQSLRREVSRLQGTGGPSQNGEGYPWQSLPEQSSDALEAWENGERS 100

Hs| NP_001185551.1 QQTELQSLRREVSRLQGTGGPSQNGEGYPWQSLPEQ-------------- 86

Hs| **ABA39072.1** QQTELQSLRREVSRLQGTGGPSQNGEGYPWQSLPEQ-------------- 86

************************************

Hs| NP_003799.1 RKRRAVLTQKQKKQHSVLHLVPINATSKDDSDVTEVMWQPALRRGRGLQA 150

Hs| NP_001185551.1 -------------QHSVLHLVPINATSKDDSDVTEVMWQPALRRGRGLQA 123

Hs| **ABA39072.1** -------------QHSVLHLVPINATSKDDSDVTEVMWQPALRRGRGLQA 123

*************************************

Hs| NP_003799.1 QGYGVRIQDAGVYLLYSQVLFQDVTFTMGQVVSREGQGRQETLFRCIRSM 200

Hs| NP_001185551.1 QGYGVRIQDAGVYLLYSQVLFQDVTFTMGQVVSREGQGRQETLFRCIRSM 173

Hs| **ABA39072.1** QGYGVRIQDAGVYLLYSQVLFQDVTFTMGQVVSREGQGRQETLFRCIRSM 173

**************************************************

Hs| NP_003799.1 PSHPDRAYNSCYSAGVFHLHQGDILSVIIPRARAKLNLSPHGTFLGFVKL 250

Hs| NP_001185551.1 PSHPDRAYNSCYSAGVFHLHQGDILSVIIPRARAKLNLSPHGTFLGFVKL 223

Hs| **ABA39072.1** PSHPDRAYNSCYSAGVFHLHQGDILSVIIPRARAKLNLSPHGTFLGFVKL 223

**************************************************
