# Supplementary figures and images for: Aberrant Expression of Functional BAFF-System Receptors by Malignant B-Cell Precursors Impacts Leukemia Cell Survival
Source: PLoS One. 2011 Jun 8;6(6):e20787. doi: 10.1371/journal.pone.0020787 (PMC3110793; doi:10.1371/journal.pone.0020787)

**Figure S2**

**
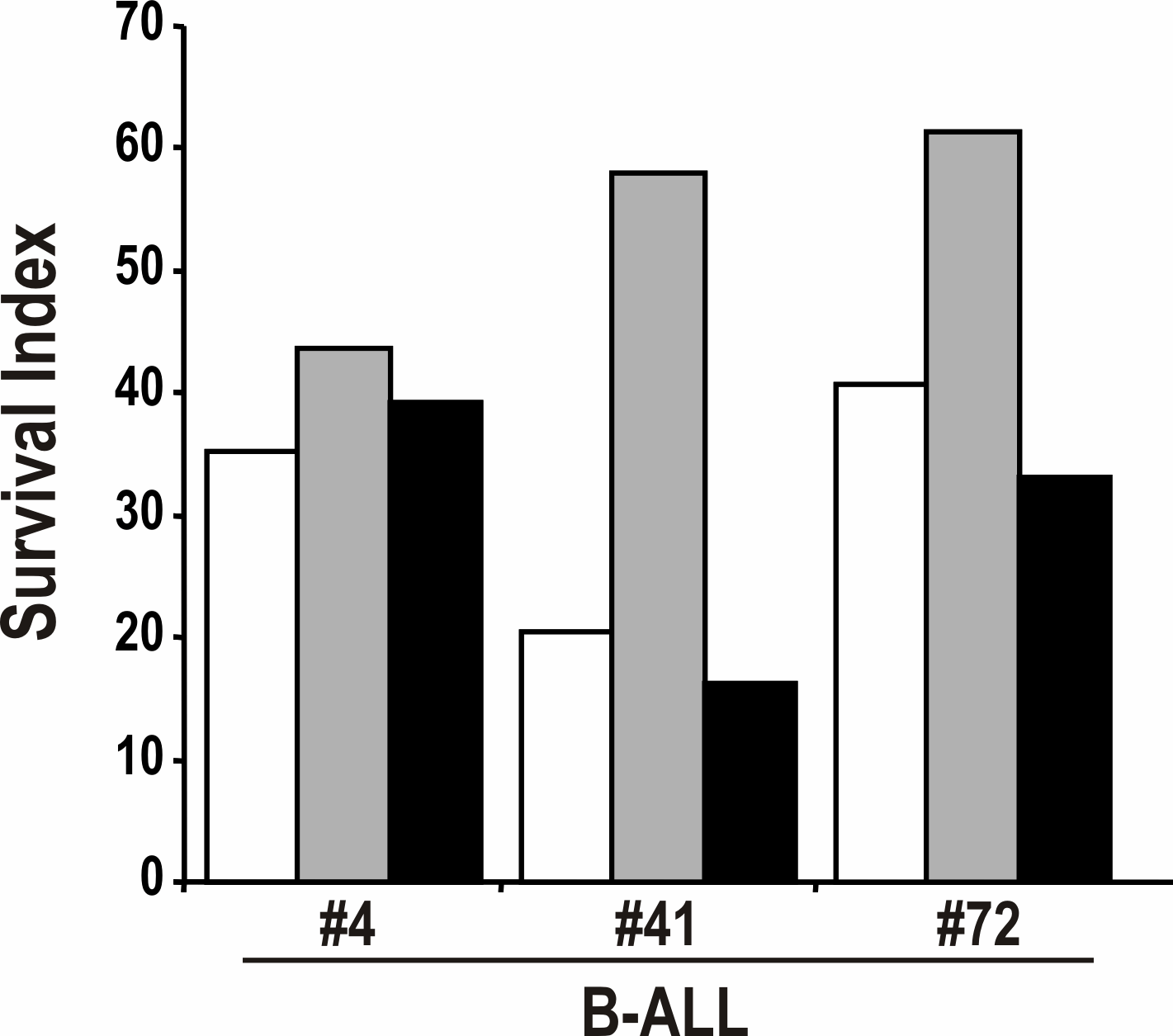
**

Supplement: Figure S2 — BCMA-Fc inhibits or abrogates the survival effect of BAFF on primary B-ALL cells. Leukemia cells (n = 3) were cultured in control medium (white bars), with BAFF-myc (100 ng/mL; gray bars) or with BAFF-myc (100 ng/mL) plus BCMA-Fc (10 µg/mL; black bars). ATP levels were quantified at 24 h and results expressed as mean Survival Index, compared to cell viability measured at day 0 (set as 100%). (DOC) [file pone.0020787.s002.doc]
