# Supplementary material for: Aberrant Expression of Functional BAFF-System Receptors by Malignant B-Cell Precursors Impacts Leukemia Cell Survival
Source: PLoS One. 2011 Jun 8;6(6):e20787. doi: 10.1371/journal.pone.0020787 (PMC3110793; doi:10.1371/journal.pone.0020787)
Supplement: Figure S3 — Genomic sequences of the APRIL gene, showing the exonic-intronic boundaries (exon 1 to exon 3). Nucleotides of exons are show in red, whereas intronic sequences are shown in black. The splicing sites donor GT and acceptor AG are indicated by the yellow or green boxes, respectively. Species abbreviations: Hs, Homo sapiens; Mm, Mus musculus. Rn, Rattus norvegicus; Bt, Bos taurus. Sequences were collected from the NCBI database, and the respective accession numbers are indicated. (DOC) [file pone.0020787.s003.doc]

**Figure S3**

Hs | NC_000017.10| ch 17

int.1 961 ctcccagaat ggggaagggt atccctggca gagtctcccg gagcaggtga gtgaggggag

1321 ttccatgagc agagttccga tgccctggaa gcctgggaga atggggagag atcccggaaa

int.2 1381 aggagagcag tgctcaccca aaaacagaag agtgaggctt ccagggtgca gcaggggtgg

1501 acagcacaac gggggaaagt ggatgcggct gagattccct ccttctctcc tcagagcagc

Mm | NC_000077.5| ch 11

int.1 481 ttcccagaag cagggagagc gcccatggca gagcctctgg gagcaggtga gtcaggggag

781 catcttagtc aaactctggc cctctttcat gagcagagtc ctgatgtcct ggaagcctgg

int.2 841 aaggatgggg cgaaatctcg gagaaggaga gcagtactca cccagaagca caagagtgag

1021 agagaagcac tcagtcctgc atcttgttcc agttaacatt acctccaagg gtaagcacta

Rn | NC_005109.2| ch 10

int.1 481 cagagcctct gggaacaggt gagttaaggg agatgggaat ctaggggtaa agggtgaatg

781 gtcaaactct ggccctcttt catgagcaga gtcctgatgt cctgggagcc tggaaggatg

int.2 841 gggccaaatc tcggagaagg agagcagtac tcacccagaa gcacaagagt gagtgagccc

1021 tagagaagca gtccgttctg catcttgttc caattaacat tacctccaag ggtaagcatt

Bt | NC_007317.4| ch 19

int.1 541 ggtgagtgag ggaagaagca tgtctggcag aaaaggatgc gtggcagggc ggtctctggg

841 ggctttctgg gacccttgca tcttagccta accctgaccc tctttccatg agcagagccc

int.2 961 ccgtaaacat aagagtgagg cctccggggt gcagcagggg tgggaggtga tccacagcag

1081 ctgctgagat ctctctctct ccccagagaa gcgctcagtt ctgcatctcg ttcccattaa
