# Supplementary material for: Aberrant Expression of Functional BAFF-System Receptors by Malignant B-Cell Precursors Impacts Leukemia Cell Survival
Source: PLoS One. 2011 Jun 8;6(6):e20787. doi: 10.1371/journal.pone.0020787 (PMC3110793; doi:10.1371/journal.pone.0020787)
Supplement: Table S1 — Clinical characteristics of B-cell ALL patients (n = 72) used in this study. Abbreviations: #, number; WBC, white blood cell count. (DOC) [file pone.0020787.s004.doc]

Table S1

| **Characteristics** | **Values** |
| --- | --- |
| ***Age, years*** |  |
| Mean | 6.48 |
| Median | 4.85 |
| Range | 0.10-17.00 |
| ***WBC, cell/μL*** |  |
| Mean | 90.95 |
| Median | 20.40 |
| Range | 2.00-1175.00 |
| ***Group Risk, # patients*** |  |
| Standard | 42 |
| High | 27 |
| Infant | 3 |
| ***Immunophenotype, # patients*** |  |
| Common | 43 |
| Pre-B | 29 |
