# Supplementary material for: Aberrant Expression of Functional BAFF-System Receptors by Malignant B-Cell Precursors Impacts Leukemia Cell Survival
Source: PLoS One. 2011 Jun 8;6(6):e20787. doi: 10.1371/journal.pone.0020787 (PMC3110793; doi:10.1371/journal.pone.0020787)
Supplement: Table S2 — Oligonucleotides and PCR conditions used for the amplification of BCMA, TACI, BAFF-R, APRIL, BAFF and PBGD. (DOC) [file pone.0020787.s005.doc]

#### Table S2

| Gene | Oligonucleotide sequence | Conditions |
| --- | --- | --- |
| *BCMA* | 5’-CACGAAGCAGGCGAAGTTCAT-3’  5’-AGTTAGAGGACAAAAAGCTGTATCGG-3’ | 60’’ at 94^o^C, 60’’ at 58^o^C, 60’’ at 72^o^C; 35 cycles |
| *TACI* | 5’-GAGCCGTGTGGACCAGGAG-3’  5’-AGGCACACACACAATGCCAAG-3’ | 60’’ at 94^o^C, 60’’ at 62^o^C, 60’’ at 72^o^C; 35 cycles |
| *BAFF-R* | 5’-GTCCCGGCCGAGTGCTT-3’  5’-GCCGGCTCCCTGCTATTGT-3’ | 45’’ at 94^o^C, 45’’ at 66^o^C, 45’’ at 72^o^C; 35 cycles |
| *APRIL* | 5’-TATTTCTCCTTGCGTAACAACCT-3’  5’-CCTTGGGACCCGAAGAGTAA-3’ | 90’’ at 94^o^C , 90’’ at 60^o^C, 90’’ at 72^o^C; 35 cycles |
| *BAFF* | 5’-CTGTCTTGCTGCCTCACGGT-3’  5’-AGAGAAAGGGAGGAAAATAGCTACA-3’ | 60’’ at 94^o^C, 60’’ at 58^o^C, 60’’ at 72^o^C; 35 cycles |
| *PBGD* | 5’-TGAGAGTGATTCGCCTGGGTAC  5’-CCCTGTGGTGGACATAGCAATG | 30” at 96^o^C, 30’’ at 55^o^C, 40’’ at 72^o^C; 35 cycles |
